# The Role of Necroptosis in Cardiovascular Disease

**DOI:** 10.3389/fphar.2018.00721

**Published:** 2018-07-06

**Authors:** Shi Zhe-Wei, Ge Li-Sha, Li Yue-Chun

**Affiliations:** ^1^Department of Cardiology, The Second Affiliated Hospital and Yuying Children’s Hospital of Wenzhou Medical University, Wenzhou, China; ^2^Department of Pediatrics, The Second Affiliated Hospital and Yuying Children’s Hospital of Wenzhou Medical University, Wenzhou, China

**Keywords:** necroptosis, vascular atherosclerosis, ischemia-reperfusion injury, cardiac remodeling, RIP1/RIP3/MLKL signaling pathway

## Abstract

A newly discovered mechanism of cell death, programmed necrosis (necroptosis), combines features of both necrosis and apoptosis. Necroptosis is tightly modulated by a series of characteristic signaling pathways. Activating necroptosis by ligands of death receptors requires the kinase activity of receptor-interacting protein 1 (RIP1), which mediates the activation of receptor-interacting protein 3 (RIP3) and mixed lineage kinase domain-like (MLKL) two critical downstream mediators of necroptosis. Recently, different cytokines have been found participating in this mechanism of cell death. Necroptosis has been proposed as an important component to the pathophysiology of heart disease such as vascular atherosclerosis, ischemia-reperfusion injury, myocardial infarction and cardiac remodeling. Targeting necroptosis signaling pathways may provide therapeutic benefit in the treatment of cardiovascular diseases.

## Introduction

According to the morphological features, there are three mechanisms of cell death have been established including necrosis, apoptosis and type 2 autophagic death (Degterev et al., 2005). Necrosis is a form of cell injury leading to the death of cells in tissues by autolysis. Apoptosis is a process of programmed cell death. Autophagy is a natural, regulated, destructive mechanism which disassembles and clears unnecessary or dysfunctional components of the cell (Levine et al., 2011).

In recent years, many scholars have proposed a new mechanism of cell death, necroptosis. Necroptosis shares some features with both apoptosis and necrosis, and activated by ligands of death receptors. It is a new form of programmed necrosis (Zhou and Yuan, 2014). Necroptosis is distinguishable from the other mechanisms because it involves active cell death triggered by specific signaling pathways rather than non-specific injury (**Table 1**; Vandenabeele et al., 2010). The activity of RIP1 and RIP3 is necessarily needed during the activation of necroptosis. The active disintegration of mitochondrial, lysosomal and plasma membranes is involved in the process of necroptosis (Vandenabeele et al., 2010).

**Table 1:** The differences between apoptosis, necrosis, and necroptosis.

Necroptosis	Necrosis	Apoptosis
Activated by ligands of death receptors	Pathological changes or non-specific injuries	Physiological or
		pathological changes
Programmed	Unprogrammed	Programmed
Release cell fragments	Release cell fragments	Phagocytosis by inflammatory cells
Cell swelling	Cell swelling	Cell shrinkage
No apoptotic bodies	No apoptotic bodies	Apoptotic bodies
Inflammatory response	Inflammatory response	No inflammatory response

Necroptosis is involved in the pathogenesis of many diseases, including neurodegeneration (Zhang et al., 2017), cancer (Meng M.B. et al., 2016), and viral infection (Orzalli and Kagan, 2017). Moreover, necroptosis also exerts an important effect on cardiovascular disease. Inhibition of programmed necrosis can reduce arterial plaque formation, alleviate ischemia-reperfusion injury, improve ventricular remodeling and so on.

In this review, the current understanding of the contributions of necroptosis to cardiovascular diseases is summarized and reviewed.

## The Regulation Factors and Signaling Pathways of Necroptosis

Typically, the cytokines such as RIP1, Fas-associated protein with death domain (FADD), Cylindromatosis (CYLD), TNF receptor-associated factor 2 (Traf2), tumor necrosis factor-α (TNF-α) and caspase 8 can activate death receptors induce apoptosis; however, in some conditions, these cytokines can also initiate necroptosis. When apoptosis is restrained or the status of caspase8 is inhibited and not optimal for apoptosis, necroptosis can be mediated by RIP1 or RIP3. RIP1 and RIP3, the characteristic proteins of necroptosis, are two crucial kinases responsible for mediating this form of cell death (He et al., 2016). A study reported that the pseudokinase, MLKL, is also the direct executioner of necroptosis (Berger et al., 2016). Both RIP3 and MLKL were specific programmed necrosis proteins.

**FIGURE 1:**
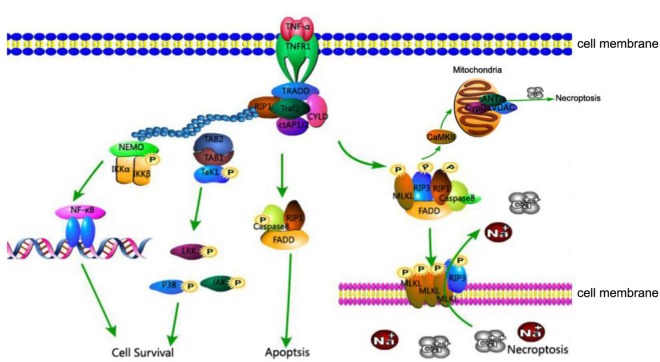
TNFR1-mediated cell death pathways of cell survival, apoptosis, and necroptosis. Association of TNFR1 with TNF trimer leads to the formation of complex I consisting of TRADD, TRAF2, RIP1, and cIAP1 at the cytoplasmic membrane. K63-linked polyubiquitination of RIP1 by cIAP1 leads to the recruitment of IKK complex and TAK1, activating the NF-κB and MAPK survival pathways. In the absence of cIAP1 or cFLIP, RIP1, FADD, and caspase-8 form cytosolic complex IIa to activate the caspase cascade and induce apoptosis. Under conditions where caspase-8 activity is inhibited, RIP1 interacts with RIP3 and MLKL to form complex IIb which is involved in mediating necroptosis. The kinase activity of RIP1 is essential to the formation of complex IIb, RIP3 and MLKL are phosphorylated in complex IIb and translocate to the plasma membrane, where the complex mediates membrane permeabilization. In addition, CaMKII, a key component of necroptosis, increases levels of ROS and leads to mitochondrial dysfunction and necrosis.

The relationship between these proteins, RIP1, RIP3, and MLKL, is as follows (**Figure 1**): The activation of death receptors further activates RIP1, which transfers and binds to RIP3 and MLKL, and forms a complex. When caspase 8 is inhibited, the RIP3-MLKL complex induces necroptosis (Zhou and Yuan, 2014).

**FIGURE 2:**
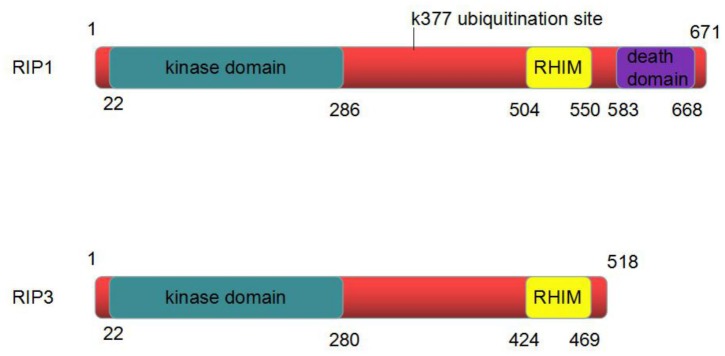
The structure of RIP1 and RIP3 protein. RIP1 is composed of 671 amino acids. It has an N-terminal kinase domain, an intermediate domain and a C-terminal death domain. RIP3 is composed of 518 amino acids. It has an N-terminal kinase domain and an intermediate domain. The intermediate domain of RIP1 contains a RIP homotypic interaction motif (RHIM) binding to the RHIM in RIP3 to activate necroptosis.

RIP1, composed of 671 amino acids, is a critical regulator of programmed necrosis. It has a C-terminal death domain (DD), an intermediate domain and an N-terminal Ser/Thr kinase domain. The DD intervenes the interplay of RIP1 with Fas and other DD-containing proteins. The intermediate domain includes a RIP homotypic interaction motif (RHIM) binding to the RHIM in RIP3 to activate necroptosis. The N-terminal Ser/Thr kinase domain and intermediate domain are both involved in mediating the activation of nuclear factor-κB (NF-κB) (**Figure 2**; Ea et al., 2006).

RIP1 is located in the cytoplasm and is widely distributed in the brain, heart, liver, kidney, spleen, small intestine, lymph and other tissues. The polyubiquitination of RIP1 at Lys-377 can be activated by TNF-α. This polyubiquitination is important for the activation of IkB kinase (IKK) and NF-κB. A point mutation of RIP1 in the area of Lys-377 (K377R) can prevent polyubiquitination and its ability to maintain the activation of IKK. The K377R mutation of RIP1 also inhibits the recruitment of transforming growth factor activated kinase-1(TAK1) and IKK complexes to the TNF receptor (Ea et al., 2006).

RIP3/RIPK3, a RIP family member, has been proven to participate in the process of necroptosis and regarded as a critical regulator of necroptosis (Moriwaki and Chan, 2013).

RIP3 is composed of 518 amino acids. RIP3 contains the same N-terminal kinase domain as RIP1; however, its C-terminal is more specific, and there is no DD. RIP3 can bind to RIP1 through the C-side shared RHIM domain (**Figure 2**). The formation of RIP1-RIP3 complex is involved in the process of programmed necrosis.

RIP3 is identified to interact with several metabolic enzymes that includes glycogen phosphorylase (PYGL), glutamate-ammonia ligase (GLUL) and glutamate dehydrogenase 1(GLUD1). These enzymes up-regulate substrates for oxidative phosphorylation, which is a major source of reactive oxygen species (ROS) in the cell (Christofferson and Yuan, 2010). Therefore, importantly, the kinase activity of RIP3 affects material metabolism and is essential for necroptosis (Newton et al., 2014).

The principal components of the necrosome are receptor-interacting protein (RIP)1 and RIP3. The intermediate domain of RIP1 contains a RIP homotypic interaction motif (RHIM) binding to the RHIM in RIP3 to compose necrosome. MLKL is another core component of the necrosome.

Mixed lineage kinase domain-like as a component of the ‘necrosome’ is consist of a four-helical bundle tethered to the pseudokinase domain, which contains an unusual pseudoactive site (Murphy et al., 2013). By interacting with RIP3, the multiprotein complex initiates TNF-induced programmed necrosis (Murphy et al., 2013).

Previous studies demonstrated that the N-terminus of MLKL, rather than the C-terminal kinase domain, is required for its function in necroptosis (Chen et al., 2014). In addition, MLKL oligomerization is also essential in necroptosis. After initiation of necroptosis, threonine 357 and serine 358 domains of the MLKL kinase are phosphorylated by RIP3. The phosphorylated MLKL is transformed from the monomeric to oligomeric state in order to activate downstream reactions and induce programmed necrosis.

Calmodulin-dependent protein kinase II (CaMKII) is abundant in the myocardium and other excitable tissues. Apart from the constitution of the RIP3/MLKL complex, RIP3 can also mediate CaMKII to induce necroptosis. Under basal conditions, CaMKII is inactivated. When intracellular Ca^2+^ rises, it calcifies calmodulin (Ca^2+^/CaM) to engage the calmodulin binding region of the regulatory domain. When a substrate binds to Ca^2+^/CaM allosterically, the catalytic domain is uninhibited by the regulatory domain, leading to CaMKII activation (Feng and Anderson, 2017).

Zhang et al. (2016) indicate that the benefits of RIP3 deficiency are mediated by preventing I/R (or H/R) and Dox induced CaMKII activation, which subsequently blocks necroptosis in myocardium.

**FIGURE 3:**
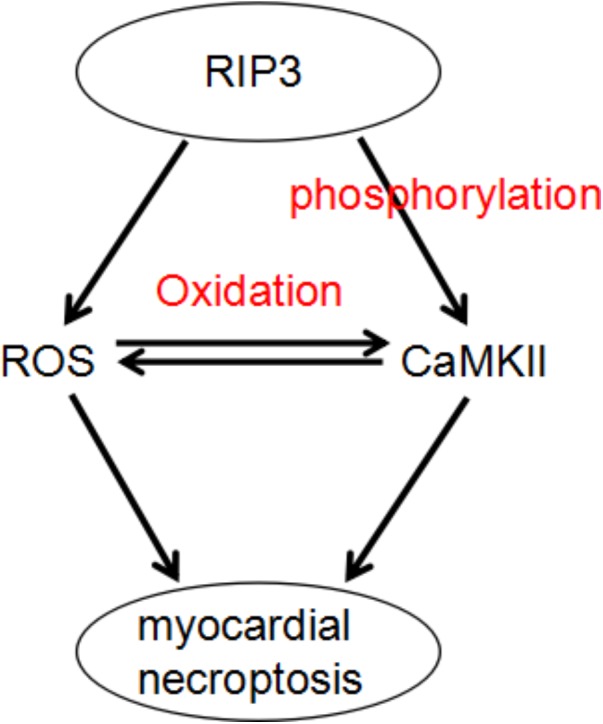
A dual mechanism that underlies RIP3-induced CaMKII phosphorylation and oxidation and subsequent myocardial necroptosis.

Recognizing CaMKII as a newly found RIP3 substrate and describing a RIP3-CaMKII myocardial necroptosis pathway may lead to identification of a perfect target for the treatment of myocardial injury and heart failing induced by ischemia and oxidative stress (**Figure 3**; Zhang et al., 2016).

Cylindromatosis is a member of the family of deubiquitinating enzymes (Trompouki et al., 2003). One important feature of the CYLD protein is that it has a specific binding site for the TNF receptor (TNFR) and NF-κB essential modulator (NEMO). Inhibition of CYLD expression inhibits TNF-α-induced Jurkat cell program necrosis, indicating that CYLD is a critical regulator of procedural necrosis (Hitomi et al., 2008).

A large number of cytokines such as caspase-8, TNF-α and Traf2 participate in the modulation of necroptosis. TNF-α-mediated signaling pathways play a key role in the initiation of programmed necrosis and stimulate induction of necroptosis.

A critical function of caspase 8 is the regulation of intestinal homeostasis and protection of IECs from TNF-α-induced necroptotic cell death (Gunther et al., 2011). Recently, Caspase-8 has also been revealed inhibiting RIP3-RIP1-kinase-dependent necroptosis following the activation of death receptor (He et al., 2009; Zhang et al., 2009).

Traf2 participated in the process of myocardial survival and homeostasis by suppressing necroptosis. Traf2 critically regulated RIP1-RIP3-MLKL necroptotic signaling with the adaptor protein TNF RI-associated Death Domain (TRADD) as an upstream regulator and TAK1 as a downstream effector (Guo et al., 2017).

Despite identification of new pathways of necroptosis, the classic pathway of programmed necrosis can be described as follows: the TNF family of cytokines and ligands of Toll-like receptors 3 and 4 can trigger the necroptosis pathway. In turn, the activated TNF receptor recruits RIP1, combining and activating a tightly associated kinase RIP3 to compose a necrosis-inducing protein complex. Marked by phosphorylation at the serine 232 site, the activated RIP3 is allowed to bind and activate its downstream effector MLKL. MLKL is then phosphorylated by RIP3. The phosphorylated MLKL shifts into an oligomerized state that facilitates the formation of membrane-disrupting pores, finally leading to necrotic death (**Figure 1**).

## The Role of Programmed Necrosis on Cardiovascular Disease

Cardiovascular disease is one of the currently leading causes of death in the world (Reamy et al., 2018). A large number of studies have proved that cell death is an integrant component in the pathogenesis of myocardial infarction, heart failure and other cardiovascular diseases (Whelan et al., 2010). Previous studies suggest that cardiomyocytes mainly die by necrosis or by apoptosis. A novel mechanism named programmed necrosis (necroptosis) was proposed as another significant mediator of cell death in cardiovascular disease (Kung et al., 2011).

## Vascular Atherosclerosis

Atherosclerosis (AS) is a chronic, lipid-driven and maladaptive inflammatory disease of the vessel wall (Xiao et al., 1997). It is one of the main causes of coronary heart disease and peripheral vascular disease.

Atherosclerosis is triggered by subendothelial retention of infiltrated low density lipoprotein (LDL) in the intimal space. Lipid metabolism dysfunction is the basis of atherosclerosis, while monocytes play an important role in the formation of atherosclerotic lesions. Monocytes can be activated by cytokines released from the area of atherosclerotic plaques. Monocytes are mobilized from the bone marrow to the lesional sites and exacerbate the formation of lesions in multiple tissues, contributing to the premature death of the animal (Meng et al., 2015).

Kavurma et al. (2017) define the different types of lesional macrophage death in AS: apoptosis, autophagic death and the newly defined necroptosis.

The necroptotic pathway is proven to be connected with vascular disease, and RIP3 and MLKL are detected within the advanced atherosclerotic plaques, supporting the view that this pathway may contribute to lesion vulnerability. Compared to normal arteries, gene expression analysis also demonstrates a significant increase in expression of both RIP3 and MLKL mRNA in atherosclerotic plaques (Karunakaran et al., 2016).

The role of RIP3 in atherosclerosis has recently been identified. RIP3 knockout mice develop less complex atherosclerotic lesions and present with reduced inflammation (Lin et al., 2013). Another study showed that mice without RIP3-mediated cell death not only displayed less severe injuries in multiplex tissues but also had obviously delayed mortality. This may be observed due to the decrease in necroptosis, which is known to be activated in lesional macrophages (Meng L. et al., 2016).

The MLKL phosphorylation, the final procedure in the execution of necroptosis, is regarded as the most definitive biomarker of necroptosis activity in atherosclerosis (Denuja et al., 2016). Phosphorylated MLKL was detected within the atherosclerotic lesions, supporting the suppose that necroptosis may contribute to lesion vulnerability.

**FIGURE 4:**
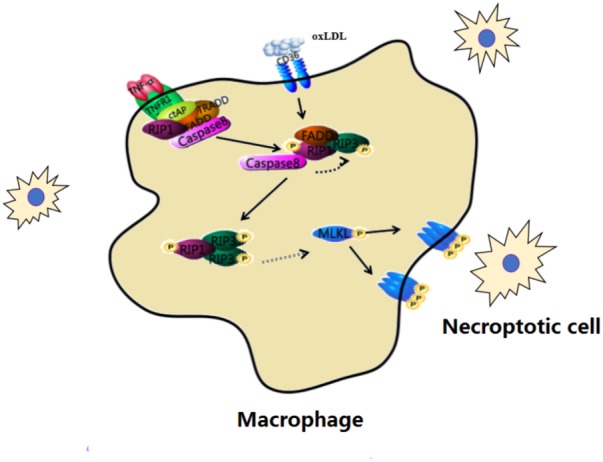
Macrophage necroptosis in atherosclerosis. In early lesions, modified lipoproteins (e.g., oxLDL) act as inflammatory stimuli within the vessel wall to recruit circulating monocytes, which avidly phagocytose these modified lipids to become macrophage foam cells. Cells then undergo necroptosis within the vessel wall.

The expression of RIP3 and MLKL is increased in humans with unstable carotid atherosclerosis. Regarded as a key step in necroptosis, MLKL phosphorylation is found in advanced atheromas. The mechanism underlying necroptosis in AS is that atherogenic forms of LDL up-regulate the transcription and phosphorylation of RIP3 and MLKL, which are two key steps in the execution of necroptosis. Activated in advanced atherosclerotic plaques, necroptotic can be targeted for both therapeutic and diagnostic interventions in experimental AS models (**Figure 4**).

In early lesions, circulating monocytes phagocytose modified lipoproteins (e.g., oxLDL) and become macrophage foam cells within the vessel wall. Then the protein expression of necroptosis such as RIP3 and MLKL are increased in macrophage foam cells. This change develops complex atherosclerotic lesions and presents inflammation. Down-regulating the process of necroptosis is beneficial to improve atherosclerosis.

## Ischemia-Reperfusion Injury

Reperfusion injury is defined as damage to tissues caused by return of blood supply after a period of ischemia or lack of oxygen (Qiao and Xu, 2016). Early literature suggests that myocardial ischemia-reperfusion (I/R) injury includes two pivotal mechanisms including necrosis and apoptosis (Skemiene et al., 2013). Necrosis is considered to be an accidental or passive type of cell death, while apoptosis is a highly modulated, genetically determined process designed to systematically eliminate damaged cells and maintain homeostasis (Freude et al., 1998).

Recently, there has been increasing evidence showing that necroptosis plays an important role in cell death during I/R injury. Koshinuma et al. (2014) isolated guinea pig hearts to investigate whether necroptosis is involved in myocardial I/R injury. In his study, by simultaneous inhibiting necroptosis and apoptosis, the reduction of myocardial infarct size was observed (Koshinuma et al., 2014). These findings provide an another therapeutic target for the improvement of left ventricular recovery after myocardial infarction.

The classic pathway of necroptosis plays an important role in I/R injury. It has been proven that RIP1/3 phosphorylation is raised after myocardial I/R injury *in vivo*. Inhibiting RIP1-mediated necrosis attenuates the myocytes initial loss as well as contributes to an increased resistance to oxidative stress and additional I/R injury, which leads to less mitochondrial dysfunction (Oerlemans et al., 2012). In addition to the well-established formation of complexes involving RIP3, RIP1 and MLKL, RIP3 can also trigger myocardial necroptosis through activation of CaMKII. CaMKII is a newly identified RIP3 substrate that helps delineate a RIP3-CaMKII-mitochondrial permeability transition pore (mPTP) myocardial necroptosis pathway, a potential therapeutic target for ischemia- and oxidative stress-induced myocardial injury and heart failure (Zhang et al., 2016).

Opening of the mPTP, a mega-channel compound in the inner mitochondrial membrane, is proved to play an important role in I/R-induced injury (Kalogeris et al., 2012; Elrod and Molkentin, 2013). RIP3 induces mPTP opening via the endoplasmic reticulum (ER) stress/calcium overload/XO/ROS pathway. RIP3 gene knockout abolished the ER stress and thus obstructed the calcium overload/XO/ROS/mPTP pathways, benefiting to a pro-survival state that leaded to the reduction of cardiomyocytes necroptosis when suffering cardiac I/R injury (Zhu et al., 2018). It suggests that necroptosis can also be induced by ER stress via the calcium overload/XO/ROS/mPTP opening axis.

The cardioprotective effects of necroptosis inhibitor necrostatin-1 are investigated on an isolated heart model in rats. Intracoronary injection of necrostatin-1 (44.5 μmol/liter) causes an increase in left-ventricular systolic pressure that produces a positive inotropic effect but does not significantly decrease the infarction zone (Dmitriev et al., 2011). Another finding shows that necrostatin-1 administration decreases RIP1/3-phosphorylation *in vivo* after I/R injury. The beneficial effect of necrostatin-1 on both short- and long-term heart function post myocardial I/R is novel (Oerlemans et al., 2012).

**FIGURE 5:**
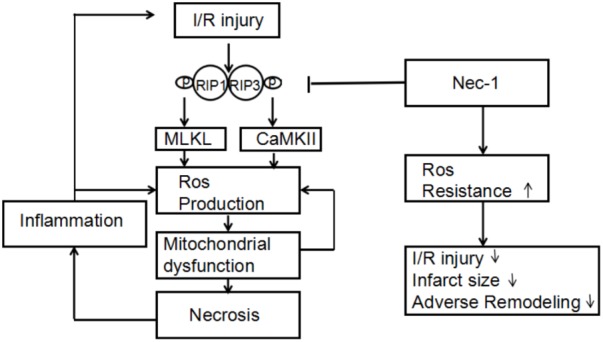
Proposed mechanism by which necrostatin-1 (Nec-1) exerts its cardioprotective effects. Upon cardiac ischemia-reperfusion (I/R), RIP1/3-mediated cell death is activated, accompanied by overgeneration of ROS in which MLKL and CaMKII are key components. Increased levels of ROS lead to mitochondrial dysfunction and necrosis, followed by the inflammatory response. Both mitochondrial dysfunction and inflammation cause an additional increase in ROS levels, triggering a chain reaction of I/R injury. Nec-1 inhibits RIP1/3 phosphorylation, thereby limiting necrotic cell death in which increased resistance to ROS seems to play an important role. Spreading of I/R injury is prevented, leading to infarct size reduction and preservation of left ventricular function.

In conclusion, myocardial I/R injury can trigger two kinds of necroptosis pathways, RIP3-CaMKII and RIP1-RIP3-MLKL (**Figure 5**). Then these necroptosis pathways take part in the initial loss of myocytes as well as possibly contributes to an increase in oxidative stress and additional I/R injury from spreading throughout the myocardium, followed by the inflammatory response. Necrostatin-1, an inhibitor of RIP1, can provide a new target to treat myocardial I/R injury. Spreading of I/R injury is prevented, leading to infarct size reduction and preservation of left ventricular function.

## Myocardial Infarction

Acute myocardial infarction is one of the main causes of death worldwide and is the end result of ischemic injury (Janahmadi et al., 2015). The necroptotic pathway plays an important role in the process of myocardial infarction.

RIP3 is expressed in cardiomyocytes and colocalizes with mitochondria. It mediates inflammation, ROS generation and adverse remodeling after myocardial infarction (Luedde et al., 2014).

Luedde et al. (2014) demonstrated that RIP3 overexpression led to a significant reduction of RIP1 protein content in neonatal rat ventricular cardiomyocytes (NRVCMs). In addition, work by Oerlemans et al. (2012) demonstrated that chemical inhibition of RIP1 after induction of ischemia leads to a reduction of infarct size (Oerlemans et al., 2012). The view of Adameova et al. (2017) on RIP1 in I/R injury leads to the same conclusion. Therefore, it is clear that the RIP1 is critically involved in the pathophysiology of myocardial infarction.

In conclusion, RIP3 mediates inflammation, ROS generation and adverse remodeling and RIP1 is critically involved in the pathophysiology of myocardial infarction, so the necroptosis pathway can be regarded as a therapeutic target for the improvement of left ventricular recovery and the reduction of myocardial infarct size after myocardial infarction. These results indicate a fundamental role of pathways mediating necroptosis in cardiac ischemia.

## Cardiac Remodeling

Ventricular size, function and shape are effected by mechanical, neurohormonal and genetic factors in the process of ventricular remodeling. Ventricular remodeling may be physiological and adaptive during normal growth or pathological due to myocardial infarction or hypertension (Sutton and Sharpe, 2000). It can lead to heart failure or even death. However, the molecular mechanisms that regulate necroptosis in heart failure and the role of necroptosis in myocardial remodeling remain largely unknown.

Li et al. (2014) put forward that the TAK1 protein, a key survival factor in the heart that directly antagonizes necroptosis, regulates myocardial remodeling. The authors indicate that, under normal conditions, ligation of TNFR1 promotes the association of TAK1 with RIP1, which prevents the formation of the RIP1-FADD-caspase 8 cell death complex. However, with low or inhibited TAK1 activity, RIP1 dissociates from TAK1 and instead binds to caspase 8 and FADD. The induction of the RIP1-FADD-caspase 8 and the RIP1-RIP3 complexes leads to necroptotic cell death. The results suggest a new model whereby caspase 8 functions as a scaffold molecule in transducing necroptotic signaling, independent of its proteolytic activity under certain cell death-inducing conditions, such as TAK1 inhibition. The authors suggest that inhibition of RIP1 may have widespread clinical utility for a range of pathological conditions involving cell death (Li et al., 2014). A recent research suggested that a Traf2-mediated and NFκB-independent prosurvival pathway can suppress necroptotic signaling. And it can serve as a therapeutic target for ventricular remodeling and heart failure (Guo et al., 2017).

Although Li lei’s and Guo’s studies clarify the role of TAK1 and Traf2 and necroptosis during ventricular remodeling clearly, more research is needed to better understand the relationship between cardiac remodeling and necroptosis to find a target gene that can delay the process of cardiac remodeling.

## Myocarditis

Basic researches have showed that necroptosis played an important role in the process of inflammation and revealed that it could be detected in the pathogenesis of a large number of inflammatory diseases (Pasparakis and Vandenabeele, 2015). Myocarditis was defined as an inflammatory disease of the myocardium. It can be diagnosed by established histological, immunological and immunohistochemical standards (Cheng et al., 2016). It is a cause of dilated cardiomyopathy and sudden cardiac death (Pollack et al., 2015).

The necroptosis pathway is not well-investigated regarding to Coxsackievirus B3 (CVB3) infection, however, according to the similarities between CVB3-induced cell lysis and necroptosis, this kind of cell death and the change of RIP1/RIP3 proteins expression may provide some insights. Zhou et al. (2017) provide evidence that RIP1/RIP3-mediated necroptosis has a great impact on cardiomyocyte death and is a crucial pathway for cell death in acute viral myocarditis.

The initial injury of myocytes induces an inflammatory response and produces a large number of inflammatory factors. The specific inflammatory factors such as TNF-α may trigger the process of necroptosis, then cause an additional increase in inflammatory response. It may be treated as an efficient therapeutic target for the treatment of acute viral myocarditis by impeding the necroptosis pathway (Zhou et al., 2017).

## The Prospects of Necroptosis Pathway

The current paper does not elaborate on how to apply the necroptosis pathway to treat heart disease. In addition to the therapeutic targets described above, the following is the progress of programmed necrosis in the treatment of heart disease in recent years.

As a small molecule capable of inhibiting a key regulator of programmed necrosis (RIP1), necrostatin-1 is demonstrated to inhibit necrotic cell death in experimental models of cardiac ischemia. The cardio-protective effect of Necrostatin-1 emphasized the significance of necrotic cell death in the ischemic heart, which opens a new direction for myocardial infarction therapy in clinic (Szobi et al., 2016).

Shen et al. (2017) suggest that Aldehyde Dehydrogenase 2 is indispensable for the favorable cardiac effect of low-to-moderate alcohol consumption and Aldehyde Dehydrogenase 2 deficiency may lead to unexpected cardiac dysfunction via enhancement of myocardial apoptosis and necroptosis pathways.

Hypercholesterolemia diet rats injected with naringenin (50 mg/kg/bw) improves all altered parameters and offers insights into a possible molecular mechanism underlying suppression of the necroptosis pathway in the heart via naringenin (Chtourou et al., 2015).

Liu J. et al. (2016) show that Ad-Hepatocyte growth factor treatment attenuates post-MI cardiac remodeling in SD rats by maintaining the heart function and minimizing the scar size and aggresome accumulation. It can improve post-MI cardiac remodeling by up-regulating autophagy and necroptosis and down-regulating apoptosis (Liu J. et al., 2016).

The 5-aminolevulinic acid-mediated sonodynamic therapy has been identified, which induces the transformation from necroptosis to apoptosis by initiating the caspase-3 and caspase-8 pathways. This therapy may ameliorate the prognosis of atherosclerosis (Tian et al., 2016).

A latest study shows that heat shock protein 70 (HSP70) reduces cardiomyocyte necroptosis by suppressing the autophagy during myocardial I/R, which reveals the novel protective mechanism of HSP70 and provides a novel molecular target for the treatment of ischemic heart disease (Liu X. et al., 2016). The beneficial effects of simvastatin pretreatment in cardiac allograft to reduce I/R injury may also relate prevention of apoptosis and necroptosis (Tuuminen et al., 2016).

The findings by Qin et al. (2016) imply that the miR-223-5p/-3p duplex’s co-operation decreases I/R-induced cardiac necroptosis at multiple levels.

The increasing evidence has implicated that inhibiting the process of necroptosis may be beneficial to treat some heart diseases. Clinical trials are needed to confirm the benefits of the therapy by inhibiting the process of necroptosis for patients with heart diseases. To date there are very few clinical studies that are conducted with the therapy by inhibiting the process of necroptosis for patients with heart diseases.

## Conclusion

RIP1, RIP3, MLKL, CYLD and other factors have been found to play an important role in the process of programmed necrosis. There is a large number of studies on ischemia-reperfusion injury and atherosclerosis showing that necroptosis increases damage to the heart. Inhibiting this mechanism of cell death reduces the damage of cardiovascular disease. These signaling pathways may represent attractive targets for future therapeutic interventions. The inhibitors targeted to signaling proteins in these necroptosis pathways have been developed to treat heart disease.

## Author Contributions

LY-C designed the experiments. SZ-W and GL-S wrote the paper.

## Conflict of Interest Statement

The authors declare that the research was conducted in the absence of any commercial or financial relationships that could be construed as a potential conflict of interest.
